# *N*-(3-Chloro­phen­yl)-2-hy­droxy­benzamide

**DOI:** 10.1107/S1600536810045046

**Published:** 2010-11-06

**Authors:** Abdul Rauf Raza, Bushra Nisar, M. Nawaz Tahir, Sumaira Shamshad

**Affiliations:** a Department of Chemistry, University of Sargodha, Sargodha, Pakistan; b Department of Physics, University of Sargodha, Sargodha, Pakistan

## Abstract

In the title compound, C_13_H_10_ClNO_2_, the dihedral angle between the aromatic rings is 5.57 (9)° and intra­molecular N—H⋯O and C—H⋯O hydrogen bonds both generate *S*(6) rings. In the crystal, mol­ecules are linked by O—H⋯O hydrogen bonds into *C*(6) chains propagating along [010]. Mol­ecules from neighbouring chains along the *z* axis are involved in C—H⋯π and π–π stacking inter­actions [centroid–centroid distance = 3.9340 (10) Å].

## Related literature

For pharmacological background to this work, see: Coupet *et al.* (1979 ▶); Pae *et al.* (2004 ▶). For related structures, see: Raza *et al.* (2009 ▶, 2010*a* ▶,*b* ▶). For graph-set notation, see: Bernstein *et al.* (1995 ▶).
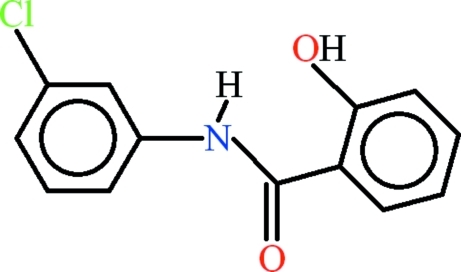


## Experimental

### 

#### Crystal data

C_13_H_10_ClNO_2_
*M* *_r_* = 247.67
Monoclinic, 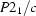

*a* = 13.4638 (5) Å
*b* = 11.9019 (4) Å
*c* = 7.1764 (2) Å
β = 98.808 (2)°
*V* = 1136.42 (7) Å^3^
*Z* = 4
Mo *K*α radiation
μ = 0.32 mm^−1^
*T* = 296 K
0.24 × 0.16 × 0.15 mm

#### Data collection

Bruker Kappa APEXII CCD diffractometer
Absorption correction: multi-scan (*SADABS*; Bruker, 2005 ▶) *T*_min_ = 0.982, *T*_max_ = 0.987
10332 measured reflections
2806 independent reflections
1827 reflections with *I* > 2σ(*I*)
*R*_int_ = 0.033

#### Refinement

*R*[*F* ^2^ > 2σ(*F* ^2^)] = 0.043
*wR*(*F* ^2^) = 0.115
*S* = 1.03
2806 reflections
161 parameters
H atoms treated by a mixture of independent and constrained refinement
Δρ_max_ = 0.25 e Å^−3^
Δρ_min_ = −0.34 e Å^−3^

### 

Data collection: *APEX2* (Bruker, 2009 ▶); cell refinement: *SAINT* (Bruker, 2009 ▶); data reduction: *SAINT*; program(s) used to solve structure: *SHELXS97* (Sheldrick, 2008 ▶); program(s) used to refine structure: *SHELXL97* (Sheldrick, 2008 ▶); molecular graphics: *ORTEP-3 for Windows* (Farrugia, 1997 ▶) and *PLATON* (Spek, 2009 ▶); software used to prepare material for publication: *WinGX* (Farrugia, 1999 ▶) and *PLATON*.

## Supplementary Material

Crystal structure: contains datablocks global, I. DOI: 10.1107/S1600536810045046/gk2313sup1.cif

Structure factors: contains datablocks I. DOI: 10.1107/S1600536810045046/gk2313Isup2.hkl

Additional supplementary materials:  crystallographic information; 3D view; checkCIF report

## Figures and Tables

**Table 1:** Hydrogen-bond geometry (Å, °) *Cg*1 is the centroid of the C1–C6 benzene ring.

*D*—H⋯*A*	*D*—H	H⋯*A*	*D*⋯*A*	*D*—H⋯*A*
O1—H1⋯O2^i^	0.88 (2)	1.73 (2)	2.6016 (18)	173 (2)
N1—H1*A*⋯O1	0.88 (2)	1.85 (2)	2.606 (2)	143.4 (18)
C13—H13⋯O2	0.93	2.30	2.869 (2)	119
C6—H6⋯*Cg*1^ii^	0.93	2.89	3.675 (2)	143

Symmetry codes: (i) 
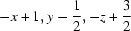
; (ii) 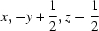.

**Fig. 1.**
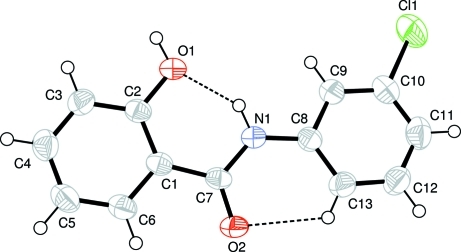
View of the title compound with the atom numbering scheme. The displacement ellipsoids are drawn at the 50% probability level. H atoms are shown by small circles of arbitrary radii. The dotted line indicates intramolecular hydrogen bond.

**Fig. 2.**
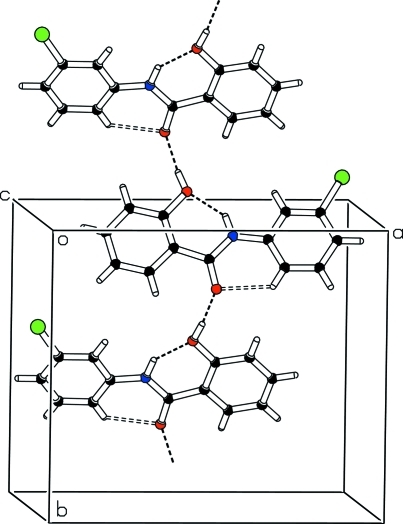
One dimensional polymeric chains via hydrogen bonds - view parallel to the b axis (PLATON; Spek, 2009).

## supplementary crystallographic information

### Comment

Benzoxazepines are known for their mild tranquilizing activities. Different synthetic derivatives of benzoxazepine are potential biological candidates and exhibit a wide range of biological activities, *e.g.*, anti-inflammatory activity (Pae *et al.*, 2004), anti-depressant and anti-psychotic activity (Coupet *et al.*, 1979). The title compound (I, Fig. 1) has been prepared as a precursor for the asymmetric synthesis of benzoxazepines.

We have reported the crystal structures of (II) *i.e.*, *N*-(4-chlorophenyl) -2-hydroxybenzamide (Raza *et al.*, 2010*a*), (III) 2-hydroxy-5-nitro -*N*-phenylbenzamide (Raza *et al.*, 2010*b*) and (IV) 2-hydroxy-3-nitro-*N*-phenylbenzamide (Raza *et al.*, 2009) which are related to the title compound. The title compound differs from (II) due to attachment of chloro group at position-3 instead of position-4.

In (I), the phenyl rings A (C1–C6) of 2-hydroxyphenyl is planar with r. m. s. deviation of 0.012 Å and the O-atom of hydroxy group [O1] is at a distance of -0.078 (2) Å. Similary the phenyl ring B (C8—C13) of 3-chloroanilinic group is planar with r. m. s. deviation of 0.004 Å and the chloro group [CL1] is at a distance of -0.076 (2) Å. The dihedral angle between A/B is 5.57 (9)°. There exist intramolecular H-bonds of N—H···O and C—H···O types (Table 1, Fig. 1) completing S(6) ring motifs (Bernstein *et al.*, 1995). The molecules are arranged to form one dimensional polymeric chains extending along the crystallographic *b* axis due to intermolecular H-bonds of O—H···O type (Table 1, Fig. 2). The C—H···π interactions (Table 1) and π–π interactions [the centroids of both aromatic rings at a distance of 3.934 (10) Å (symmetry: 1 - *x*, - *y*, 1 - *z)]* play an important role in stabilization of the crystal.

### Experimental

To a well stirred solution of 2-hydroxybenzoic acid (1.38 g, 0.01 mol, 1 eq) and SOCl_2_ (0.87 ml, 1.42 g, 0.012 mol, 1.2 eq) in dry CHCl_3_, 3-chloroaniline (1.05 ml, 1.27 g, 0.01 mol, 1 eq) and Et_3_N (2.08 ml, 1.5 g, 0.015 mol, 1.5 eq) was added slowly at room temperature, followed by reflux for three hours. After the completion of the reaction, the reaction mixture was cooled to room temperature, neutralized with aqueous NaHCO_3_ (10%) and the title compound was obtained as a white solid. The crude solid was filtered off and recrystallized from CHCl_3_ to afford white prisms.

### Refinement

The coordinates of H atoms of the amide and hydroxy groups were refined whereas the remaining H atoms were positioned geometrically with C–H = 0.93 Å and were included in the refinement in the riding model approximation. The isotropic displacement parameters of H atoms were set as *U*_iso_(H) = 1.2*U*_eq_(C, N, O).

### Crystal data

**Table:**

C_13_H_10_ClNO_2_	*F*(000) = 512
*M**_r_* = 247.67	*D*_x_ = 1.448 Mg m^−^^3^
Monoclinic, *P*2_1_/*c*	Mo *K*α radiation, λ = 0.71073 Å
Hall symbol: -P 2ybc	Cell parameters from 1827 reflections
*a* = 13.4638 (5) Å	θ = 1.5–28.5°
*b* = 11.9019 (4) Å	µ = 0.32 mm^−^^1^
*c* = 7.1764 (2) Å	*T* = 296 K
β = 98.808 (2)°	Prism, white
*V* = 1136.42 (7) Å^3^	0.24 × 0.16 × 0.15 mm
*Z* = 4	

### Data collection

**Table:**

Bruker Kappa APEXII CCD diffractometer	2806 independent reflections
Radiation source: fine-focus sealed tube	1827 reflections with *I* > 2σ(*I*)
graphite	*R*_int_ = 0.033
Detector resolution: 7.5 pixels mm^-1^	θ_max_ = 28.5°, θ_min_ = 2.3°
ω scans	*h* = −17→17
Absorption correction: multi-scan (*SADABS*; Bruker, 2005)	*k* = −15→15
*T*_min_ = 0.982, *T*_max_ = 0.987	*l* = −5→9
10332 measured reflections	

### Refinement

**Table:**

Refinement on *F*^2^	Primary atom site location: structure-invariant direct methods
Least-squares matrix: full	Secondary atom site location: difference Fourier map
*R*[*F*^2^ > 2σ(*F*^2^)] = 0.043	Hydrogen site location: inferred from neighbouring sites
*wR*(*F*^2^) = 0.115	H atoms treated by a mixture of independent and constrained refinement
*S* = 1.03	*w* = 1/[σ^2^(*F*_o_^2^) + (0.0498*P*)^2^ + 0.1662*P*] where *P* = (*F*_o_^2^ + 2*F*_c_^2^)/3
2806 reflections	(Δ/σ)_max_ = 0.001
161 parameters	Δρ_max_ = 0.25 e Å^−^^3^
0 restraints	Δρ_min_ = −0.34 e Å^−^^3^

### Special details

**Table:**

Geometry. Bond distances, angles *etc*. have been calculated using the rounded fractional coordinates. All su's are estimated from the variances of the (full) variance-covariance matrix. The cell e.s.d.'s are taken into account in the estimation of distances, angles and torsion angles
Refinement. Refinement of *F*^2^ against ALL reflections. The weighted *R*-factor *wR* and goodness of fit *S* are based on *F*^2^, conventional *R*-factors *R* are based on *F*, with *F* set to zero for negative *F*^2^. The threshold expression of *F*^2^ > σ(*F*^2^) is used only for calculating *R*-factors(gt) *etc*. and is not relevant to the choice of reflections for refinement. *R*-factors based on *F*^2^ are statistically about twice as large as those based on *F*, and *R*- factors based on ALL data will be even larger.

### Fractional atomic coordinates and isotropic or equivalent isotropic displacement parameters (Å^2^)

**Table:**

	*x*	*y*	*z*	*U*_iso_*/*U*_eq_	
Cl1	0.95784 (4)	−0.09691 (5)	0.80925 (10)	0.0858 (3)	
O1	0.49322 (9)	−0.04858 (10)	0.79519 (19)	0.0502 (3)	
H1	0.4719 (16)	−0.1099 (18)	0.840 (3)	0.071 (7)*	
O2	0.55912 (9)	0.26044 (9)	0.57224 (17)	0.0480 (3)	
N1	0.61841 (11)	0.09050 (11)	0.6738 (2)	0.0414 (3)	
H1A	0.5995 (13)	0.0264 (16)	0.714 (2)	0.050*	
C1	0.44202 (12)	0.13109 (13)	0.6707 (2)	0.0365 (4)	
C2	0.41952 (12)	0.02934 (13)	0.7532 (2)	0.0389 (4)	
C3	0.32279 (13)	0.00851 (15)	0.7912 (2)	0.0461 (4)	
H3	0.3090	−0.0579	0.8505	0.055*	
C4	0.24770 (14)	0.08575 (16)	0.7414 (3)	0.0522 (5)	
H4	0.1834	0.0714	0.7677	0.063*	
C5	0.26700 (13)	0.18469 (15)	0.6526 (3)	0.0536 (5)	
H5	0.2155	0.2358	0.6160	0.064*	
C6	0.36320 (13)	0.20707 (14)	0.6187 (2)	0.0452 (4)	
H6	0.3760	0.2741	0.5600	0.054*	
C7	0.54346 (12)	0.16594 (13)	0.6345 (2)	0.0367 (4)	
C8	0.72051 (13)	0.10029 (13)	0.6548 (2)	0.0390 (4)	
C9	0.78234 (13)	0.01425 (15)	0.7347 (2)	0.0456 (4)	
H9	0.7562	−0.0433	0.8002	0.055*	
C10	0.88287 (14)	0.01472 (16)	0.7165 (3)	0.0522 (5)	
C11	0.92390 (15)	0.09982 (18)	0.6235 (3)	0.0589 (5)	
H11	0.9920	0.1001	0.6141	0.071*	
C12	0.86155 (14)	0.18490 (16)	0.5443 (3)	0.0547 (5)	
H12	0.8883	0.2427	0.4802	0.066*	
C13	0.76052 (13)	0.18621 (14)	0.5579 (2)	0.0467 (4)	
H13	0.7196	0.2439	0.5030	0.056*	

### Atomic displacement parameters (Å^2^)

**Table:**

	*U*^11^	*U*^22^	*U*^33^	*U*^12^	*U*^13^	*U*^23^
Cl1	0.0602 (4)	0.0860 (5)	0.1122 (5)	0.0296 (3)	0.0165 (3)	0.0241 (4)
O1	0.0486 (7)	0.0319 (7)	0.0723 (9)	0.0043 (6)	0.0163 (6)	0.0114 (6)
O2	0.0565 (8)	0.0283 (6)	0.0615 (8)	0.0036 (5)	0.0166 (6)	0.0055 (5)
N1	0.0423 (8)	0.0303 (7)	0.0532 (9)	0.0016 (6)	0.0123 (6)	0.0043 (6)
C1	0.0434 (9)	0.0304 (8)	0.0350 (8)	0.0017 (7)	0.0033 (7)	−0.0061 (6)
C2	0.0437 (9)	0.0322 (8)	0.0402 (9)	0.0015 (7)	0.0044 (7)	−0.0051 (7)
C3	0.0465 (10)	0.0411 (10)	0.0506 (10)	−0.0058 (8)	0.0076 (8)	−0.0008 (8)
C4	0.0391 (10)	0.0557 (12)	0.0611 (11)	−0.0035 (8)	0.0056 (8)	−0.0098 (9)
C5	0.0431 (10)	0.0474 (11)	0.0666 (12)	0.0093 (8)	−0.0040 (9)	−0.0048 (9)
C6	0.0463 (10)	0.0362 (9)	0.0507 (10)	0.0044 (8)	−0.0010 (8)	−0.0024 (7)
C7	0.0456 (9)	0.0273 (8)	0.0370 (8)	0.0027 (7)	0.0062 (7)	−0.0049 (6)
C8	0.0426 (9)	0.0351 (9)	0.0404 (9)	0.0006 (7)	0.0095 (7)	−0.0055 (7)
C9	0.0472 (10)	0.0415 (10)	0.0495 (10)	0.0040 (8)	0.0122 (8)	0.0022 (8)
C10	0.0467 (10)	0.0532 (11)	0.0563 (11)	0.0094 (9)	0.0065 (8)	−0.0018 (9)
C11	0.0426 (10)	0.0674 (13)	0.0679 (13)	−0.0031 (10)	0.0125 (9)	−0.0082 (10)
C12	0.0542 (11)	0.0509 (11)	0.0617 (12)	−0.0099 (9)	0.0174 (9)	−0.0004 (9)
C13	0.0507 (10)	0.0394 (10)	0.0508 (10)	−0.0028 (8)	0.0105 (8)	0.0000 (8)

### Geometric parameters (Å, °)

**Table:**

Cl1—C10	1.7381 (19)	C4—H4	0.9300
O1—C2	1.3579 (19)	C5—C6	1.380 (2)
O1—H1	0.87 (2)	C5—H5	0.9300
O2—C7	1.2400 (19)	C6—H6	0.9300
N1—C7	1.348 (2)	C8—C9	1.387 (2)
N1—C8	1.407 (2)	C8—C13	1.391 (2)
N1—H1A	0.866 (18)	C9—C10	1.380 (2)
C1—C6	1.401 (2)	C9—H9	0.9300
C1—C2	1.401 (2)	C10—C11	1.375 (3)
C1—C7	1.488 (2)	C11—C12	1.381 (3)
C2—C3	1.393 (2)	C11—H11	0.9300
C3—C4	1.373 (2)	C12—C13	1.379 (2)
C3—H3	0.9300	C12—H12	0.9300
C4—C5	1.383 (3)	C13—H13	0.9300
			
C2—O1—H1	112.7 (14)	O2—C7—N1	121.10 (15)
C7—N1—C8	129.47 (14)	O2—C7—C1	121.81 (14)
C7—N1—H1A	114.0 (12)	N1—C7—C1	117.09 (14)
C8—N1—H1A	116.5 (12)	C9—C8—C13	119.72 (16)
C6—C1—C2	117.81 (15)	C9—C8—N1	115.60 (14)
C6—C1—C7	116.86 (14)	C13—C8—N1	124.65 (15)
C2—C1—C7	125.34 (14)	C10—C9—C8	119.55 (17)
O1—C2—C3	120.55 (15)	C10—C9—H9	120.2
O1—C2—C1	119.08 (15)	C8—C9—H9	120.2
C3—C2—C1	120.37 (15)	C11—C10—C9	121.47 (18)
C4—C3—C2	120.22 (16)	C11—C10—Cl1	119.74 (15)
C4—C3—H3	119.9	C9—C10—Cl1	118.78 (15)
C2—C3—H3	119.9	C10—C11—C12	118.42 (18)
C3—C4—C5	120.50 (17)	C10—C11—H11	120.8
C3—C4—H4	119.7	C12—C11—H11	120.8
C5—C4—H4	119.7	C13—C12—C11	121.54 (18)
C6—C5—C4	119.56 (17)	C13—C12—H12	119.2
C6—C5—H5	120.2	C11—C12—H12	119.2
C4—C5—H5	120.2	C12—C13—C8	119.29 (17)
C5—C6—C1	121.45 (16)	C12—C13—H13	120.4
C5—C6—H6	119.3	C8—C13—H13	120.4
C1—C6—H6	119.3		
			
C8—N1—C7—O2	−0.4 (3)	C1—C2—C3—C4	2.6 (3)
C8—N1—C7—C1	−179.66 (16)	C2—C3—C4—C5	0.2 (3)
C7—N1—C8—C9	170.31 (17)	C3—C4—C5—C6	−1.8 (3)
C7—N1—C8—C13	−11.9 (3)	C4—C5—C6—C1	0.7 (3)
C6—C1—C2—O1	176.28 (15)	N1—C8—C9—C10	177.43 (18)
C6—C1—C2—C3	−3.6 (2)	C13—C8—C9—C10	−0.4 (3)
C7—C1—C2—O1	−4.1 (2)	N1—C8—C13—C12	−178.15 (19)
C7—C1—C2—C3	176.04 (16)	C9—C8—C13—C12	−0.5 (3)
C2—C1—C6—C5	2.0 (3)	C8—C9—C10—Cl1	−177.25 (16)
C7—C1—C6—C5	−177.67 (17)	C8—C9—C10—C11	1.4 (3)
C2—C1—C7—O2	−175.16 (15)	Cl1—C10—C11—C12	177.29 (17)
C2—C1—C7—N1	4.1 (2)	C9—C10—C11—C12	−1.3 (3)
C6—C1—C7—O2	4.5 (2)	C10—C11—C12—C13	0.4 (3)
C6—C1—C7—N1	−176.25 (15)	C11—C12—C13—C8	0.5 (3)
O1—C2—C3—C4	−177.32 (17)		

### Hydrogen-bond geometry (Å, °)

**Table:**

Cg1 is the centroid of the C1–C6 benzene ring

**Table:**

*D*—H···*A*	*D*—H	H···*A*	*D*···*A*	*D*—H···*A*
O1—H1···O2^i^	0.88 (2)	1.73 (2)	2.6016 (18)	173 (2)
N1—H1A···O1	0.88 (2)	1.85 (2)	2.606 (2)	143.4 (18)
C13—H13···O2	0.93	2.30	2.869 (2)	119
C6—H6···Cg1^ii^	0.93	2.89	3.675 (2)	143

Symmetry codes: (i) −*x*+1, *y*−1/2, −*z*+3/2; (ii) *x*, −*y*+1/2, *z*−1/2.
